# Advanced Manufacturing of PLA Surgical Templates for Orbital Floor Geometry: Optimizing Fidelity and Surface Morphology via Variable Layer Height MEX 3D Printing

**DOI:** 10.3390/ma19061208

**Published:** 2026-03-19

**Authors:** Paweł Turek, Grzegorz Budzik, Łukasz Przeszłowski, Anna Bazan, Bogumił Lewandowski, Paweł Pakla, Tomasz Dziubek, Robert Brodowski, Małgorzata Zaborniak, Jan Frańczak, Michał Bałuszyński

**Affiliations:** 1 Department of Manufacturing Techniques and Automation, Rzeszów University of Technology, 35-959 Rzeszów, Poland; abazan@prz.edu.pl; 2 Department of Mechanical Engineering, Rzeszów University of Technology, 35-959 Rzeszów, Poland; gbudzik@prz.edu.pl (G.B.); lprzeszl@prz.edu.pl (Ł.P.); tdziubek@prz.edu.pl (T.D.); mzab@prz.edu.pl (M.Z.); 3 Department of Cranio-Maxillofacial Surgery, University of Rzeszów, 35-315 Rzeszów, Poland; boglewandowski@wp.pl (B.L.); robert.brodowski@wp.pl (R.B.); 4 Department of Maxillofacial Surgery, University Clinical Hospital Fryderyk Chopin in Rzeszów, 35-055 Rzeszów, Poland; pawel.pakla@gmail.com (P.P.); janek.franczak@gmail.com (J.F.);

**Keywords:** additive manufacturing, TARMM procedure, PLA material, surface roughness, geometrical accuracy, surgical template, orbital floor

## Abstract

**Highlights:**

- Precision and Validation: The TARMM procedure ensures orbital floor geometry accuracy up to 0.1 mm, which has been clinically validated across 21 medical cases.
- VLH Optimization: Implementing Variable Layer Height (0.07 mm for critical details and 0.3 mm for the base) eliminates stair-step artifacts while maintaining manufacturing speed.
- Surface Quality: A strong correlation (r = 0.99) between layer thickness and roughness allows for the smoothness necessary for precise contouring of titanium meshes.
- Safety and Cost-Effectiveness: The method provides an economical and non-toxic alternative to expensive systems based on photopolymer resins.
- Operational Efficiency: Reducing model preparation time to 30 min enhances operating theatre efficiency and minimizes the risk of intraoperative errors.
- Surgical Standard: The TARMM workflow is a scalable solution ready for implementation as a routine standard in maxillofacial surgery.

**Abstract:**

Precise orbital floor reconstruction requires personalised surgical templates that combine high geometric fidelity with manufacturing efficiency. This study presents and validates the TARMM procedure, developed to optimise the production of polylactide (PLA) templates. A key innovation is the integration of advanced machine learning algorithms (Random Forest) and Mitchell–Netravali interpolation to reduce medical reconstruction artefacts, as well as the implementation of Material Extrusion (MEX) technology with Variable Layer Height (VLH). This strategy minimises the stair-step effect on complex anatomical curvatures while maintaining high process throughput. The results demonstrate that the TARMM procedure ensures a geometric error within ±0.1 mm. A strong linear correlation (r = 0.99) was found between layer height and surface roughness (Sa), indicating that a 0.07 mm layer in critical areas significantly improves template morphology and facilitates the contouring of titanium meshes. The clinical validation across 21 cases confirmed a 30 min reduction in surgical preparation time. The developed method serves as a low-cost, high-precision alternative to photopolymerization technologies, contributing to modern 3D printing applications in maxillofacial surgery.

## 1. Introduction

Orbital fractures account for approximately 10–25% of all maxillofacial fractures, most frequently affecting men in their 30s and 40s as a result of direct mechanical trauma [1]. The orbital floor, the most commonly damaged structure, requires precise reconstruction and is often accompanied by other fractures of the craniofacial skeleton [2,3]. Although autologous bone grafts were historically the standard, contemporary surgery increasingly relies on engineering materials such as titanium [4,5,6], polyethene composites [7,8], and biodegradable scaffolds (HA, PTMC, PDLA) [9,10,11]. A key challenge in reconstructing such complex anatomy is intraoperative implant contouring. Surgical success often depends on the surgeon’s ability to recreate the three-dimensional bone structure manually; however, without appropriate auxiliary tools, this process carries a risk of error [5,7,12,13]. Implant fitting inaccuracies can lead to functional complications, such as diplopia [14], underscoring the need for advanced planning methods.

The contemporary approach is based on reverse engineering and the digital modelling of anatomical structures derived from tomographic data [15,16,17,18]. Due to the unique geometry of the defects, these models are ideal candidates for AM [19,20,21]. In the context of producing surgical templates, MEX technology is gaining prominence due to its cost-effectiveness [22,23] and the availability of biocompatible materials, such as PLA [24,25,26,27]. However, the application of MEX technology for the production of precise orbital templates faces significant limitations regarding geometric fidelity and surface morphology [28,29]. This process, based on layer-by-layer material deposition [30,31], is inherently susceptible to the stair-stepping effect [32,33]. This is particularly problematic for the thin-walled structures of the orbital floor (0.74–1.5 mm [34,35]), where standard layer resolution may fail to capture the fluidity of anatomical curvatures. These errors, compounded by the anisotropic structure of Digital Imaging and Communications in Medicine (DICOM) data and imaging artefacts [36,37,38], directly translate to the surface quality of the StreoLiTography (STL) model and, consequently, the physical print [39,40].

In the standard MEX process, the manufacturing accuracy of a PLA template is strongly correlated with layer height [41]. This parameter defines a compromise: thinner layers improve surface morphology and geometric fidelity but drastically increase production time and cost [42,43]. Conversely, thicker layers accelerate the process but produce a rougher surface, hindering precise contouring of titanium meshes [44,45,46,47,48]. In the case of templates used for bending implants [6,7,8], poor surface quality can lead to inaccurate mesh adaptation, potentially nullifying the effort invested in digital planning. Therefore, optimising the manufacturing process of surgical templates necessitates going beyond standard 3D printing parameters. This study proposes a solution to this problem by applying the VLH technique to MEX printing. This approach allows for the adaptive adjustment of print resolution: increasing precision in areas with complex curvature (to maintain high surface morphology) while accelerating printing in geometrically less critical sections.

While traditional CT-based reconstruction workflows in maxillofacial surgery have significantly improved surgical precision, they often struggle with the partial volume effect when segmenting ultra-thin (0.5–1.0 mm) cortical bone structures, such as the orbital floor [49]. Standardised semi-automatic segmentation [50] and Constant Layer Height (CLH) printing [51] further exacerbate these limitations by introducing a pronounced staircase effect on shallowly angled, curved surfaces. This creates a critical bottleneck: achieving high topological fidelity requires extremely thin layers, which exponentially increase manufacturing time and the risk of thermal degradation of the polymer [52]. Recent research highlights a significant lack of consistency in the accuracy of standard MEX technology. While some studies report mean deviations of approximately 0.15 mm–0.2 mm [53], others, such as [54], indicate that errors can reach as high as 0.48 mm for FFF-printed models. This discrepancy demonstrates that the precision of MEX technology is heavily dependent on specific process parameters and geometric complexity. In the confined space of the orbit, such sub-millimetre inaccuracies are clinically significant, as they may lead to improper implant positioning, enophthalmos, or diplopia.

Consequently, the implementation of the TARMM procedure and VLH strategies, as proposed in this study, is essential for ensuring the high topological fidelity required in maxillofacial surgery. The true novelty of this research lies in the synergistic integration of the TARMM workflow. Unlike conventional approaches, our method combines advanced Mitchell–Netravali image interpolation and Machine Learning-based (Random Forest) segmentation with VLH manufacturing, specifically tailored for orbital floor PLA templates. This novelty is further underscored by a comprehensive validation strategy. By bridging high-precision optical metrology—at both macro and micro scales—with clinical validation involving 21 patient cases, we provide a holistic assessment of the workflow’s fidelity. Reflecting its innovative character and practical utility, this method has been granted patent protection in the Republic of Poland (Pat. 247185).

## 2. Materials and Methods

**Figure 1:**
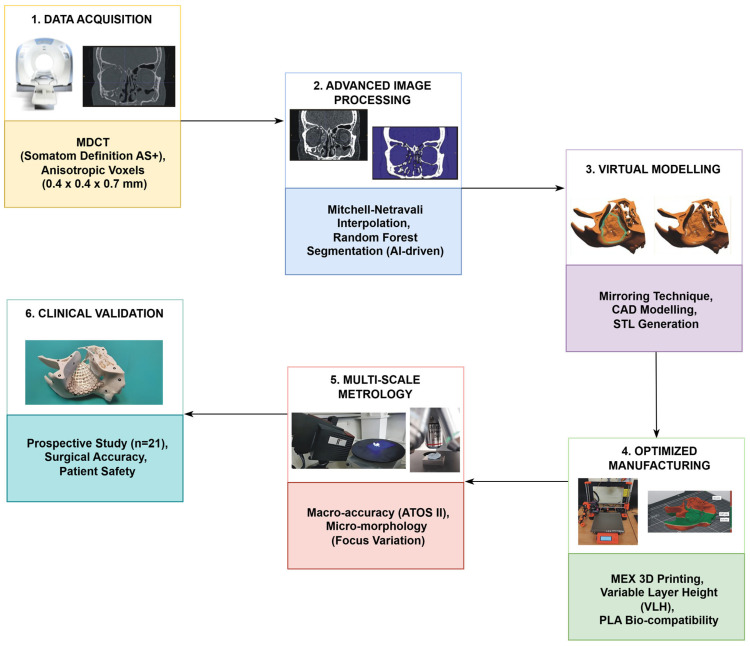
Schematic workflow of the TARMM procedure integrating stages from medical data acquisition to clinical application.

The integrated research and implementation process was structured according to the original TARMM workflow. As shown in the schematic (Figure 1), this methodology encompasses six key stages: MDCT data acquisition, advanced image processing using Mitchell–Netravali interpolation and Random Forest-based segmentation, CAD of surgical templates, MEX additive manufacturing with VLH optimisation, multi-scale metrological validation (macro and micro), and prospective clinical validation.

Between 2022 and 2023, a prospective clinical study was conducted through a collaboration between the Department of Maxillofacial Surgery at the Fryderyk Chopin University Clinical Hospital in Rzeszów and the Rzeszów University of Technology. The study cohort comprised 21 male patients (Figure S1). The inclusion criteria were adult male patients aged 20–70; diagnosed with an isolated orbital floor fracture requiring surgical intervention; availability of high-quality MDCT imaging; and who had signed informed consent. The exclusion criteria included: multi-wall orbital fractures; previous orbital surgery on the affected side; general contraindications to surgical procedures under general anaesthesia; and lack of patient consent for data publication. The research protocol was approved by the Bioethics Committee of the Medical College at the University of Rzeszów (Resolution No.2/B/2018) and the Bioethics Committee of the Medical Board in Rzeszów, in accordance with the 1964 Declaration of Helsinki and its subsequent amendments. All participants provided informed, written consent for the use of their clinical data and medical imaging—including photographs and radiographs—for scientific and publication purposes. Diagnostic testing was performed using a Somatom Definition AS+ multidetector tomograph, with a focus on the middle craniofacial region. The scanning utilised a helical protocol with tube settings of 120 kV and Care Dose4D 250 mAs. Collimation was 40 mm × 0.6 mm, the acquired slice width was 1.0 mm, and the reconstructed slice width was 0.7 mm. The matrix size was 512 × 512. Although the protocol produced high-resolution DICOM data, the resulting voxel dimensions were anisotropic (0.4 mm × 0.4 mm × 0.7 mm), which complicated the segmentation and reconstruction of the orbital floor geometry.

### 2.1. The Reconstruction Process of Orbital Geometry

**Figure 2:**
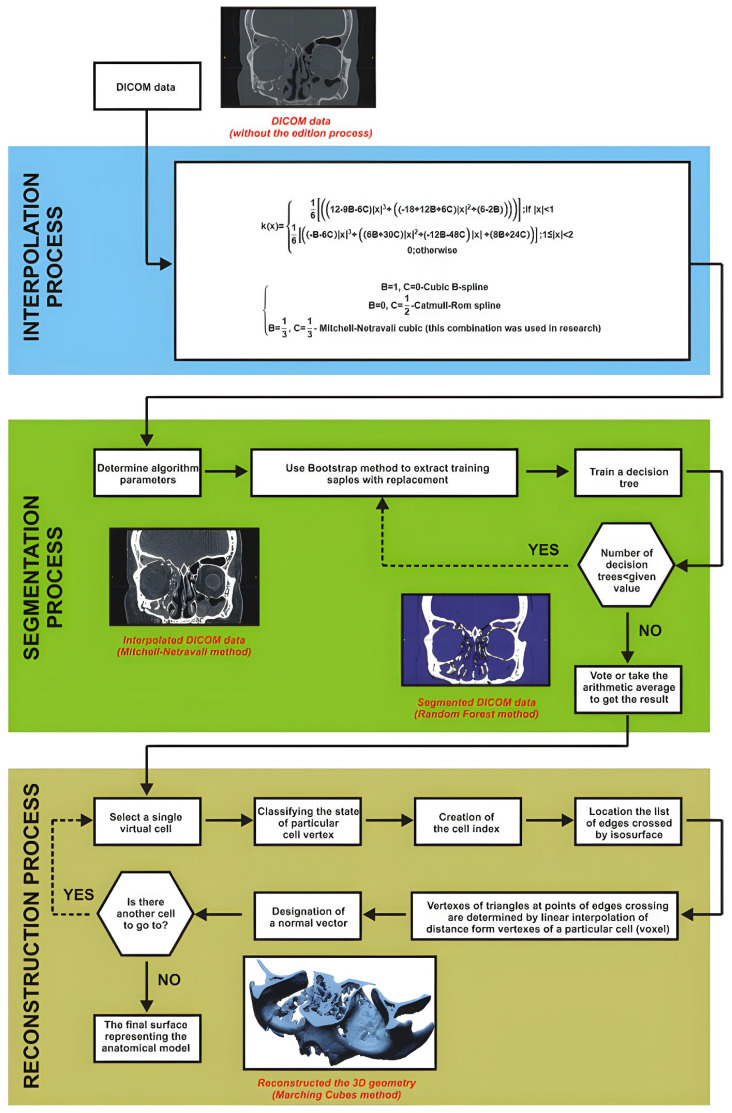
Applied procedure for the reconstruction of the geometry of the middle craniofacial region.

The first stage of the procedure aims to enhance DICOM data processing and improve the accuracy of 3D reconstruction of the central craniofacial region (Figure 2). In this stage, the initial step involves the interpolation of DICOM data using the Mitchell–Netravali algorithm [55,56]. The cubic filter was configured with coefficients B = 1/3 and C = 1/3 to optimise the transition between grayscale values while minimising reconstruction artefacts. This method utilises a four-pixel neighbourhood to generate additional pixels based on grayscale values, thereby restoring information lost during digitisation and reducing partial volume averaging artefacts in 2D images. Subsequently, a segmentation process of the central craniofacial region is performed using a Random Forest machine learning approach [57,58] comprising 100 individual decision trees. The model was trained on a dataset of 6300 voxels manually annotated across the 21 clinical cases (300 points per dataset) to represent bone and non-bone structures. To segment the middle craniofacial area, two distinct regions are marked to define the orbital bone structure and its surrounding areas. This is achieved by selecting pixels within a specific grayscale range. These selected areas serve as input for the classification algorithm, which utilises an ensemble of decision trees. The final result is a binarisation of the 2D image into two distinct regions, effectively delineating the orbital floor and adjacent structures. The segmentation performance was evaluated using 5-fold cross-validation, yielding a Dice Similarity Coefficient (DSC) of 0.94, confirming the high reliability of the automated threshold estimation. The three-dimensional anatomical model is visualised using the Marching Cubes method [59]. The space is partitioned into a series of cubes (voxels), and the nodes of each cube are evaluated against a predefined iso-value determined during the segmentation stage. Polygons corresponding to the iso-values passing through these points are then generated within the cubes. Finally, the 3D model is obtained and exported as an STL file. During the export process, the chordal deviation was set to 0.005 mm and the angular deviation to 5°. These parameters were selected to minimise geometric approximation errors during data conversion.

### 2.2. The 3D-CAD Modelling Process of the Surgical Template

**Figure 3:**
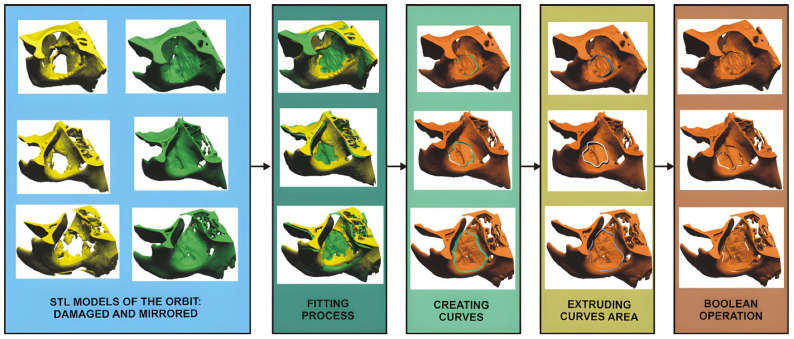
The CAD modelling procedure for the surgical template proposed in the article was presented for the three selected patients.

The second stage of the procedure involves utilising Computer Aided Design (CAD) modelling functions (as shown in Figure 3) to develop a three-dimensional model of the damaged orbit based on the reconstructed central craniofacial region. In parallel, a reference model is generated by mirroring the healthy orbital structure onto the affected side. Given the inherent asymmetry of the human body, simple mirroring is often insufficient for precise anatomical alignment. To address this, the article proposes the Best-fit method, a standard approach for aligning complex geometric structures [60,61]. This is an iterative process that minimises the sum of squared distances between the two models. The implementation requires an alignment accuracy of 0.01 mm; if the deviation exceeds this threshold, the iteration continues until the required precision is achieved. Once the models are aligned, a spline curve is traced along the boundary of the damaged area. Two additional auxiliary curves are then generated, symmetrically offset by 0.5 mm. These three curves are projected onto a plane, and a parametric surface is used to approximate the defect area. An extension of up to 2 mm is applied at the intersection with the mirrored model of the orbital floor, followed by a “digital cut” executed via Boolean operations. This process allows for precise visualisation and isolation of the extent of the damage. The final model is saved in STL format, serving as the geometric foundation for AM processes.

### 2.3. Manufacturing Surgical Templates Using an MEX Process

**Figure 4:**
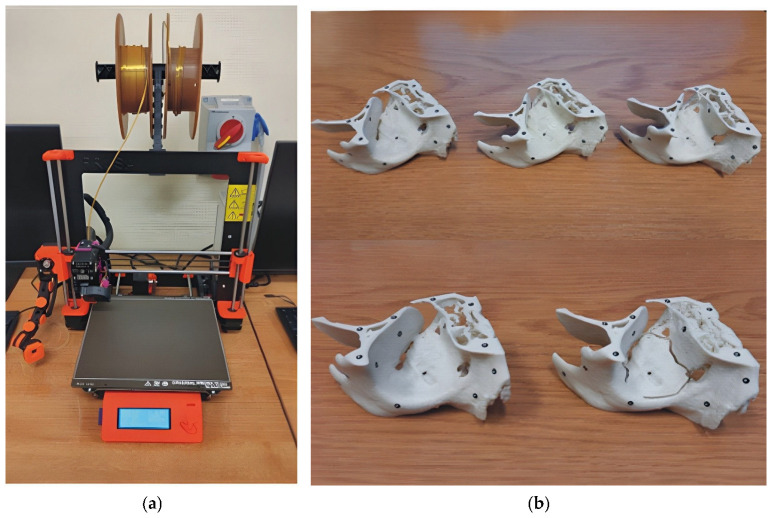

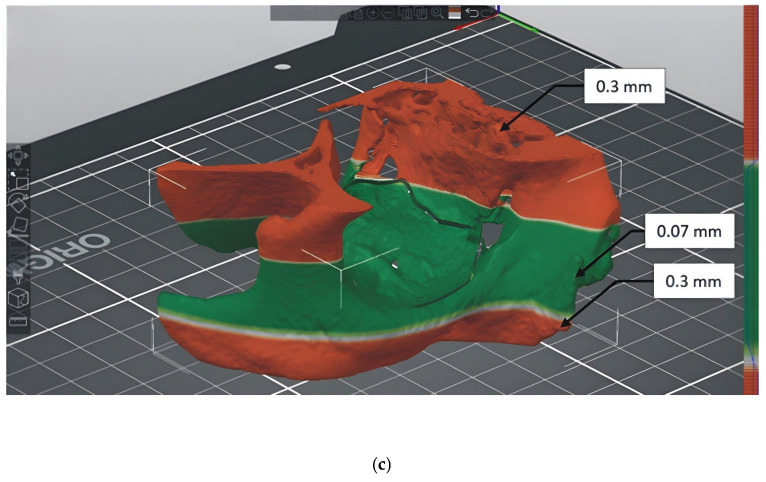
The MEX process of model manufacture: (**a**) Prusa MK3s printer; (**b**) manufactured surgical templates (after clean-up of support material) designed based on the tomographic data of patient number 10; (**c**) The View of PrusaSlicer software (version 2.5.0) during the preparation of the template model indicated variable layer 0.3 mm/0.07 mm/0.3 mm.

The MEX process was employed to manufacture surgical template models of the orbital region. The 3D printing process involved melting the filament within a heated extrusion head and precisely depositing it onto a building platform. Following the deposition of the initial layer, subsequent layers are added until the model’s full geometry is achieved [30,42]. The manufacturing was performed using a Prusa MK3s printer (Figure 4a). Prusament PLA (Prusa Polymers, Prague, Czech Republic) was selected as the fabrication material. PLA used in this study is a linear aliphatic polyester derived from renewable resources, specifically corn starch [62]. The synthesis process of the utilised filament involves several key stages: the hydrolysis of corn starch into glucose, followed by microbial fermentation to produce lactic acid. The final polymer chains are formed via polymerisation (typically ring-opening polymerisation of lactide) [56,57]. The macromolecular structure of the PLA is defined by the repeating unit [–O–CH(CH_3_)–CO–]_n_. The chemical architecture consists of a carbon backbone with integrated ester groups (–COO–) and pendant methyl groups (–CH_3_). The polymer exhibits a predominantly linear chain morphology with minimal branching. The presence of these polar ester bonds renders the material susceptible to hydrolysis, the primary mechanism of its biodegradation—a critical feature for its potential biomedical applications [63]. In terms of physical state, the PLA filament and the resulting 3D-printed models exhibit a predominantly amorphous or low semi-crystalline structure. This is a direct consequence of the thermal kinetics during both filament extrusion and the MEX process, where rapid cooling (quenching) prevents the ordered arrangement of polymer chains into crystalline lamellae. From a manufacturing perspective, this amorphous state is highly advantageous as it significantly reduces processing shrinkage, minimises internal thermal stresses, and enhances inter-layer adhesion (neck growth), thereby improving the dimensional stability and fidelity of the printed models. For research purposes, mirrored orbital models were manufactured using both constant layer heights (0.3 mm, 0.2 mm, and 0.07 mm) and a variable layer height strategy (0.3 mm/0.07 mm/0.3 mm) (Figure 4b). Figure 4c illustrates the section of the model produced with variable layer thickness. The objective of this approach was to achieve high geometric fidelity in the critical orbital floor area with a thinner layer (0.07 mm), while using a thicker layer (0.3 mm) in less essential regions to optimise production. Two types of models—one with and one without the visualisation of the orbital damage range—were manufactured to evaluate and compare the effects of variable layer thickness on the final output.

The PrusaSlicer software utilises the VLH tool to prepare the numerical data for manufacturing models with varying layer thicknesses. This feature also enables automatic smoothing of transitions between different layer heights across the model geometry. The first step in preparing the numerical data is to select a general layer-height profile. When implementing variable layer heights, specific contours in the preview must be identified where the output value is to be increased or decreased. The regions affected by these changes are highlighted in distinct colours in the model preview (Figure 4c). When defining a variable layer height, it is crucial to adhere to the layer-height limits dictated by the nozzle diameter. In this study, a 0.4 mm nozzle was used. The comprehensive 3D printing parameters are presented in Table 1.

### 2.4. Macro- and Micro-Geometry Measurements of Surgical Templates

**Figure 5:**
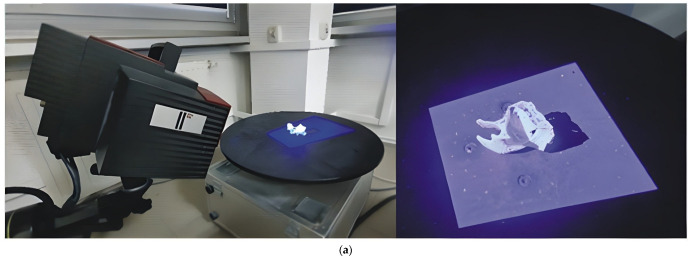

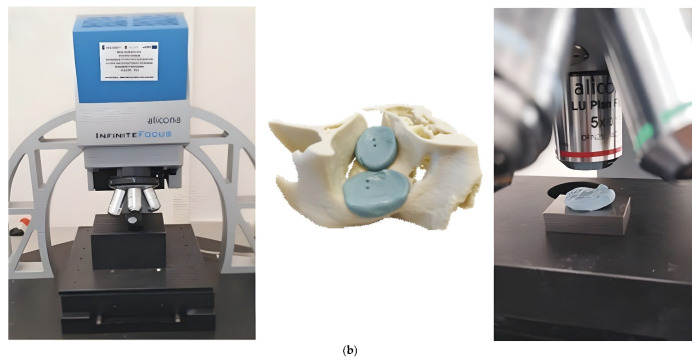
Geometry and surface roughness measurement: (**a**) Atos II Triple Scan system; (**b**) InfiniteFocus G4 microscope.

The ATOS II Triple Scan structured light scanner was employed to evaluate the macro-geometric accuracy of the manufactured templates (Figure 5a). This optical system is ideally suited for verifying the geometric precision of objects with complex morphologies [47,61,64]. The measurement setup consists of a scanning head mounted on a tripod—equipped with a projector and two six-megapixel cameras—a rotary table, and a computer system for data processing. The scanner acquires surface geometry data through the projection of Gray code stripes. The implementation of blue light technology minimizes the influence of ambient lighting conditions and significantly reduces acquisition time. To ensure high metrological standards, the system was calibrated using a certified ceramic calibration plate (SO 170) following the VDI/VDE 2634 Part 3 [65] guidelines for optical 3D measuring systems. The calibration deviation was maintained below 0.010 pixels, which, for the utilized measuring volume (170 × 130 × 130 mm^3^), corresponds to an Estimated Measurement Uncertainty (U) of approximately 0.025 mm (at a 95% confidence level, k = 2). To ensure repeatability, each template was scanned three times under stable lighting and temperature conditions (21 ± 1 °C). The resulting point clouds were then processed to generate a mean deviation map, with a standard deviation across measurements not exceeding 0.012 mm. The measurement station was prepared by placing reference points on the rotary table. All surgical template models were prepared identically: reference points were applied to their surfaces, and they were coated with a thin layer of anti-reflective spray (chalk). To enhance process efficiency and eliminate the need for manual repositioning of the scanned object, an automated measurement sequence was adopted. Based on preliminary tests, it was determined that 16 steps (rotational positions) were optimal for a complete reconstruction of the template geometry. This procedure ensured full coverage of the anatomical structures, and the resulting data were exported as a three-dimensional point cloud in STL format

Surface topography was characterized using the Alicona InfiniteFocus G4 microscope (Figure 5b) for one random clinical case (Grambach, Austria). This instrument utilizes the Focus Variation method, which relies on analyzing the focal plane of a surface image within an optical system to determine the height of surface irregularities [66,67]. The system is equipped with an array of objectives that allow for measurements at various lateral and vertical resolutions. The complete surface geometry is reconstructed through sequential vertical scanning, aggregating focused data from regions that may initially fall outside the depth of field. For every region of the object, an in-focus image is captured at a specific vertical scanner position. The acquired data is then processed to generate a high-resolution three-dimensional topographic model. Due to the optical properties of the models—specifically their translucency and reflectivity—topography measurements were performed on surface replicas. These replicas were produced using the Struers RepliFix-2 silicone-based compound to ensure high-fidelity transfer of the surface features (Ballerup, Denmark). Measurements of the surface topography were performed using an InfiniteFocus G4 focus-variation microscope manufactured by Alicona (Grambach, Austria). Due to the optical properties of the models, the measurements were carried out on replicas made with RepliFix-2 produced by Struers. For each model, three surface fragments were measured, each with dimensions of 4 mm × 5.3 mm. Measurements on different models were performed approximately at the same locations. The measurement parameters were as follows: objective magnification ×5, vertical resolution 2 µm, lateral resolution 7.8 µm, and pixel size 1.75 µm × 1.75 µm. The analysis of the measurement data was performed using IFM 3.5.1.5 (evaluation software for the InfiniteFocus microscope, Raaba, Austria) and SPIP 6.4.2. In IFM 3.5.1.5, using built-in algorithms and based on visual assessment, filters removing the form and waviness components of the surface were selected. For this purpose, a high-pass filter with a cut-off wavelength of 1 mm was applied. Filtering and further analysis were then performed in SPIP 6.4.2. Visual inspection of the printed models revealed clearly visible layers of deposited material, which influence not only the surface micro-geometry but also the overall reproduced shape of the orbital anatomy. Due to the limited resolution of the measurement instrument, primarily intended for macro-geometry assessment, parameters capable of capturing surface variations related to the applied layer thickness were required. In this case, features typically interpreted as surface roughness or waviness may also influence the reproduced anatomical shape. To characterize the surface geometrical structure, the following parameters were selected: Sa (arithmetical mean height of the scale-limited surface), the sum of Spk (reduced peak height), Sk (core height), and Svk (reduced dale height), and Rsm (mean profile spacing). The S parameters were determined for the entire measured surface, whereas the Rsm parameter for a given surface was determined from six profiles approximately perpendicular to the observed material layers. The Sa parameter represents an averaging height parameter, whereas the sum Spk + Sk + Svk reflects the overall vertical range of surface features. In optical measurements, artifacts in the form of spikes—unusually high peaks and deep valleys—are often visible. These spikes can distort the analysis when the Sz parameter (maximum height of the scale-limited surface) is used. Similar information can instead be obtained from the sum Spk + Sk + Svk, which is less sensitive to spike artifacts. The Rsm parameter was used to characterize the spatial distribution of surface features associated with the layered structure produced during printing. Lower Rsm values correspond to smaller spacing between features and are therefore perceived as a smoother surface. In the investigated models, the effects of layer thickness on macro-geometry (shape) and surface topography (micro-geometry) cannot be clearly separated, because the magnitude of surface irregularities classified as surface texture may also contribute to deviations in the reproduced shape. For this reason, these surface texture parameters were included in the analysis.

## 3. Results and Discussion

Designing and manufacturing anatomical models, implants, or specialised tools for implant contouring presents significant engineering challenges [68,69,70]. This process requires a multidisciplinary synergy between medical and technical sciences. Integrating these fields enables the development of robust procedures for the digital reconstruction of anatomical structures, followed by their physical realisation via 3D printing. Today, such solutions are increasingly prevalent, as they allow surgeons to enhance preoperative planning and surgical precision. This translates directly into reduced general anaesthesia time, minimised blood loss, and a lower risk of intraoperative complications [19,71,72,73].

**Figure 6:**
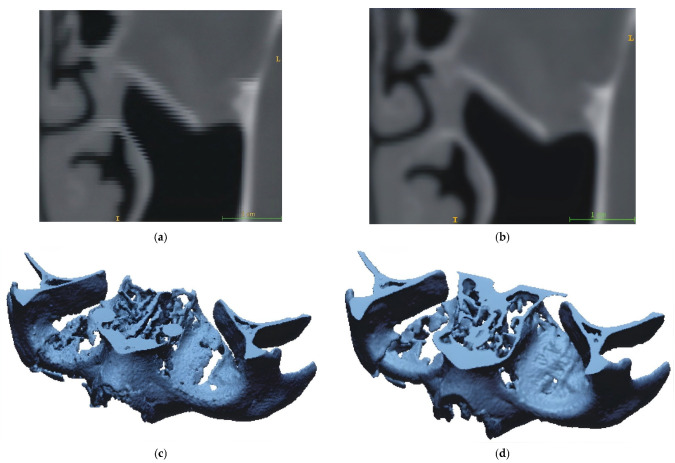
Visualization of 2D data and 3D-STL model: (**a**) DICOM data without processing; (**b**) After applying the Mitchell–Netravali algorithm; (**c**) 3D model after using thresholding segmentation process; (**d**) 3D model after using random forest segmentation process.

Developing customised orbital implants—tailored to the specific anatomy of the eye socket—is a promising approach to improving clinical outcomes and wound healing [12,74]. While CAD systems allow for the precise modification of implant thickness during the design phase, the process is often hampered by difficulties in reconstructing the complex orbital geometry [74,75,76]. These issues primarily arise during the digitisation and processing of DICOM data obtained from multidetector computed tomography (MDCT) [5,13,74]. Such data often exhibit an anisotropic voxel structure, in which uneven voxel dimensions lead to blurred object boundaries and discontinuities in the reconstructed anatomical contours. These phenomena are attributed to partial volume averaging artefacts (Figure 6a) and stair-step artefacts—distortions that occur during the conversion of volumetric data into 3D geometry [77,78]. Although high-resolution protocols and thin-slice scanning can mitigate these errors, limitations imposed by ethics committees often restrict significant adjustments to diagnostic parameters. The primary objective remains the protection of patient health by minimising exposure to ionising radiation [79].

The research presented in this article focuses on enhancing the spatial resolution of existing DICOM data to minimise the impact of partial volume averaging. The Mitchell–Netravali algorithm was implemented to balance image blurring and edge sharpening, thereby increasing the spatial resolution of 2D images in the orbital floor region. This algorithm also facilitated achieving geometric edge continuity (Figure 6b). The segmentation of bony structures within the orbital floor geometry is particularly challenging due to three factors:

- The exceptionally thin and complex anatomy of the bone.
- The technical limitations inherent in multidetector tomography.
- The subjective nature of selecting segmentation thresholds can lead to unintended geometric alterations.

While the thresholding method is standard for imaging bone structures [37,39,40], it possesses significant drawbacks (Figure 6c). Manual segmentation is often required to fill surface gaps, introducing the risk of human error. Similarly, Adaptive Thresholding does not always guarantee accurate boundary determination [80]. This article proposes a novel approach utilising the Random Forest method to achieve more accurate segmentation threshold estimation. This technique relies on an averaged iso-value derived from a study of 21 patients and employs a decision tree during binarisation. Consequently, a complete and precise visualisation of the orbital floor geometry was achieved by combining Mitchell–Netravali interpolation with machine-learning-based segmentation (Figure 6d).

The Marching Cubes algorithm was employed to reconstruct the geometry into a 3D STL model. This method is a standard approach to reconstructing volumetric data [81]. However, its limitations are well documented; the primary challenge in generating a triangle mesh from DICOM data is noise in 2D images. This noise often results in artefacts—extraneous mesh areas that do not belong to the analysed object. Furthermore, the quality of the transition from segmented contours to a faceted surface depends heavily on the slice thickness used in tomographic imaging. In cases of an anisotropic voxel structure, voids in the triangle mesh and the stair-step effect may occur. These issues arise from insufficient DICOM data density, which complicates the smooth connection of adjacent contours. Incorporating the Mitchell–Netravali algorithm into the procedure significantly mitigated these issues. The interpolation process produced a complete triangle mesh by minimising stair-step artefacts. It is also important to note that structural errors in the mesh are frequently observed when using the Marching Cubes method. The most prevalent errors include

- Incorrect triangle orientation (inverted normals);
- Duplicated edges and vertices;
- Overlapping or duplicated triangles.

Consequently, it is crucial to perform a thorough mesh repair and preparation process to eliminate these topological errors before proceeding to 3D printing.

Due to the high risk of postoperative complications and the stringent precision required during orbital surgeries, 3D printing techniques are gaining increasing popularity [4,5,74]. In surgical planning, CAD mirroring is conventionally used to create two templates representing the damaged and undamaged sections of the orbit, respectively [4,11]. This approach was adopted during the initial stages of collaboration between Rzeszów University of Technology and the Fryderyk Chopin University Clinical Regional Hospital No. 1 in Rzeszów. The MEX process was employed to manufacture the geometry of the orbital anatomical structures, as it is currently the most prevalent method in this field [6,12]. A key challenge in the medical industry remains the development of solutions that reduce production costs and lead times [82,83,84,85]. To minimise manufacturing time, a constant layer thickness of 0.3 mm was initially implemented. However, this decision led to difficulties during the surgical planning stage; surgeons reported issues with accurately contouring the implant geometry. This was attributed to the prominent layered texture on the model surfaces, resulting from the anisotropic voxel structure of the DICOM data combined with the chosen layer thickness. To address these issues, a proprietary procedure known as TARMM was developed as part of the collaboration. Instead of producing two separate orbital models, a single-model solution was devised, enabling simultaneous assessment of the damaged area and precise formation of the implant geometry. This approach is unprecedented in the literature, and the entire manufacturing procedure has been filed with the Patent Office of the Republic of Poland.

Table 2 presents the averaged results for 21 patients, detailing the manufacturing times and material costs estimated using PrusaSlicer software. For research purposes, parameters were simulated for damaged orbital models using a constant layer thickness of 0.3 mm. A comparison between the Model_0.07 and Model_0.3/0.07_0.3 variants demonstrated that implementing variable layer height significantly reduced both production costs and manufacturing time. It should be noted, however, that although adaptive layer-height algorithms offer clear economic benefits, they must be applied judiciously, considering the model’s geometry and the 3D printer’s technical capabilities.

Zeiss Inspect software was utilized to verify the geometric accuracy of the manufactured models. The alignment between the nominal model, obtained during the RE/CAD phase, and the reference model, generated through measurements using the Atos II Triple Scan optical system (GOM GmbH, Braunschweig, Germany), was performed using the best-fit method with an accuracy of 0.005 mm. A tolerance range of ±0.2 mm was established for the verification process.

The analysis results include

**Figure 7:**
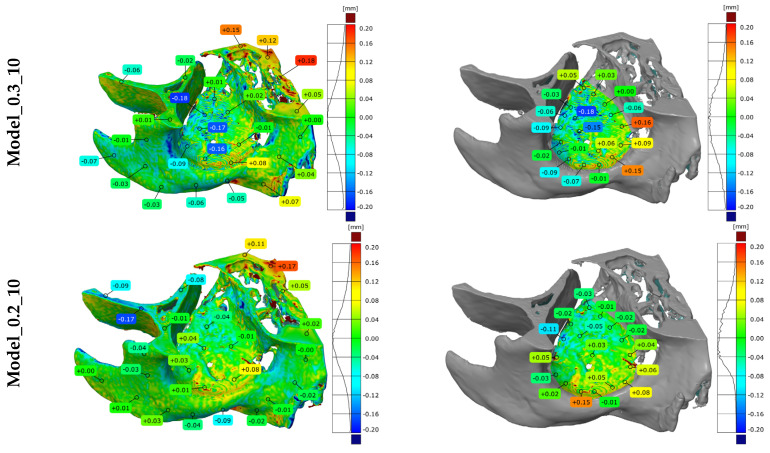

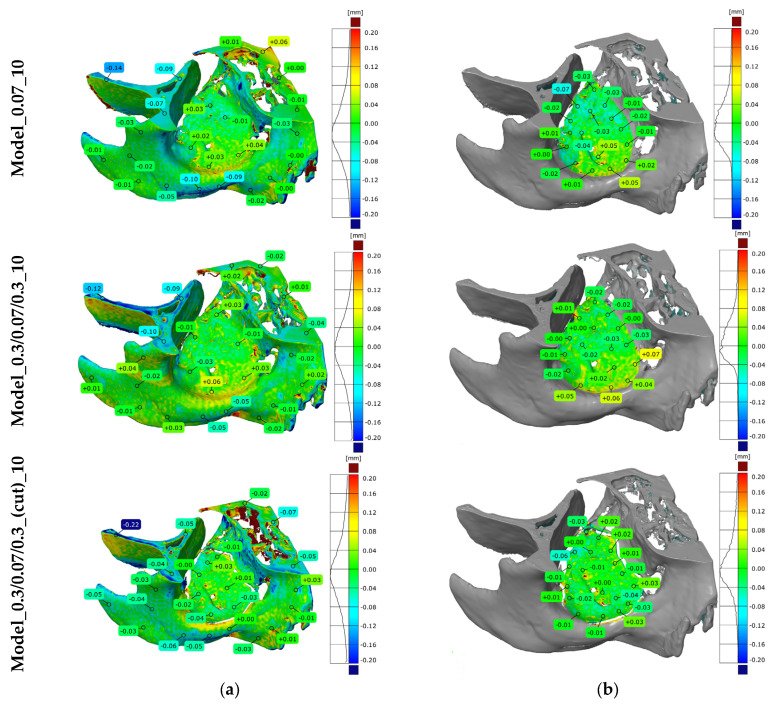
Three-dimensional deviation maps obtained from measurements on the Atos II Triple Scan for patient No. 10 for: (**a**) The entire orbital model; (**b**) A fragment of the orbital floor geometry.

- Three-dimensional deviation maps for the selected patient.
- Histogram data distribution for the entire orbital model (Figure 7a);
- A segment of the orbital floor geometry is used as the basis for forming the implant (Figure 7b).

Table 3 summarises the statistical parameters used to assess the accuracy of the orbital geometry and the specific fragment on which the implant was contoured (bent). The most significant deviations, both positive and negative, were localised at the model edges or in areas characterised by a high curvature gradient. Fortunately, most of these inaccuracies were outside the critical orbital floor area. Quantitative data in Table 3 provide a direct baseline for the manufacturing phase. The results confirmed that an increased layer thickness exacerbates the model’s stair-step effect. Interestingly, the mean and standard deviation values were consistent across all baseline models produced with a constant layer thickness (0.2 mm and 0.3 mm). However, for the orbital floor geometry, the Model_0.07 and the optimised VLH variant demonstrated measurably more favourable averaged deviation values than the baseline CLH models. This confirms that the variable layer-height strategy in the TARMM workflow actively drives morphological accuracy rather than simply masking defects.

This study breaks new ground by demonstrating high manufacturing accuracy, with results falling well within the acceptable tolerance of ±0.25 mm [86,87]. Direct comparison with existing literature remains challenging due to the scarcity of research regarding the geometric accuracy of orbital models produced via AM. However, when benchmarked against studies of other craniofacial structures, the deviations observed in this work were significantly lower. Specifically, tolerances in the critical orbital floor area remained within ±0.1 mm, underscoring the high potential and innovation of this novel approach [53,54]. It is important to note that the achieved accuracy of ±0.1 mm in critical areas exceeds the baseline nominal CT voxel resolution (0.4 mm × 0.4 mm × 0.7 mm), providing a clear quantitative measure of the algorithmic improvement. This sub-voxel precision is not merely a result of physical smoothing during manufacturing, but is fundamentally driven by the initial digital processing stages. Specifically, compared to standard baseline methods, the Mitchell–Netravali interpolation increases information density, and the Random Forest segmentation demonstrates high quantitative fidelity with a DSC of 0.94. As highlighted in Figure 6, this AI-driven approach overcomes the structural gaps inherent to baseline thresholding segmentation. Therefore, the error budget is tightly controlled from acquisition to the final manufacturing resolution (0.07 mm layer height in critical areas).

**Figure 8:**
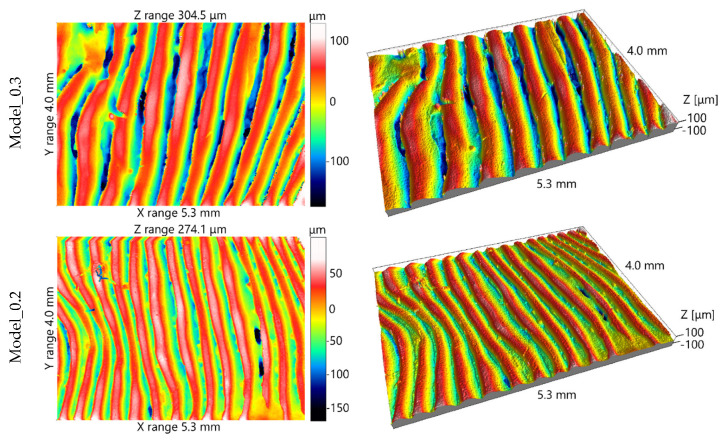

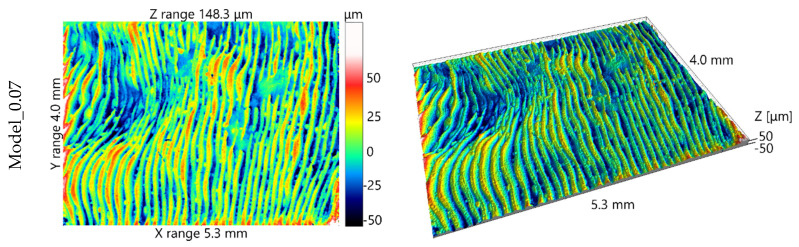
2D and 3D pseudo-colour views of 4 mm of 5.3 mm surface fragments measured in area C of models with different layer thicknesses.

Analysis of surface topography was performed based on surface texture data acquired using an InfiniteFocus microscope. Figure 8 presents 2D and 3D surface views in pseudo-colours. In all models, the individual layers of the bonded material are clearly visible. It was observed that layer morphology varies depending on the model; for instance, the surfaces of models with layer thicknesses of 0.2 mm and 0.3 mm exhibit greater visual similarity than those of the 0.2 mm and 0.07 mm variants.

**Figure 9:**
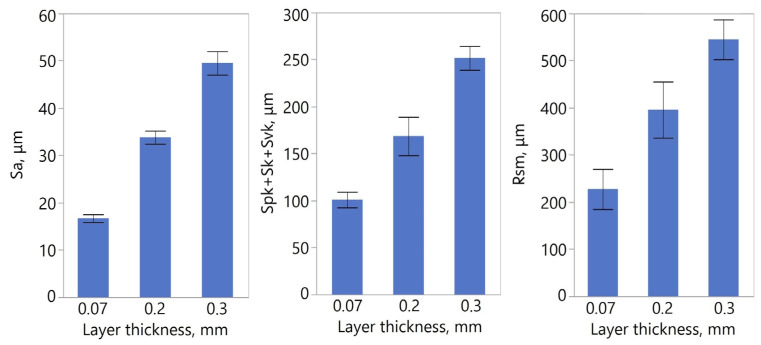
Structure parameters of samples with different layer thicknesses.

Table 4 summarises the calculated surface texture parameters. Figure 9 shows plots illustrating the effect of layer thickness on selected surface texture parameters. A strong positive correlation was observed between the layer thickness and the investigated parameters (Table 5). The Pearson correlation coefficient (r) between layer thickness and the Rsm parameter was 0.97, and between layer thickness and the sum of Spk + Sk + Svk was 0.99. These values, which are nearly equal to 1, indicate a robust linear relationship between the variables analysed. Although the number of samples was limited (n = 9), the very high correlation coefficient and the logical consistency of the observed relationship suggest a strong association between the variables analysed. The high geometric fidelity achieved in the orbital floor models is attributed to the low crystallinity of the PLA grade used, which resulted in minimal thermal contraction during the transition from the melt to the solid state.

According to previous studies [88,89,90], increasing the layer thickness in the MEX process using PLA leads to higher amplitude parameters such as Ra, Sa, and Sz. These values also depend heavily on the model’s orientation and its position within the 3D printer’s build volume. Work [88] reported Ra values ranging from 4 µm to 19 µm. In study [89], the Sa parameter ranged from 25 µm to 29 µm for a flat orientation—which most closely aligns with the conditions in this study—while Sz values ranged from 131 µm to 171 µm. The results obtained in this paper can only be compared with these findings approximately, given differences in experimental conditions, particularly in surface orientation during manufacturing. The results obtained in this paper can only be compared with these findings approximately, given differences in experimental conditions, particularly in surface orientation during manufacturing. Building on this comparison with alternative manufacturing routes, it is important to note that the TARMM workflow aims to democratise the production of surgical templates by utilising FDM technology, which is significantly more cost-effective than resin-based systems such as SLA or DLP [53]. While SLA/DLP printers typically achieve superior surface finishes, with layer heights often below 50 μm, and better material isotropy, the literature suggests that the clinical requirement for surgical guide fit often falls within a 0.2–0.5 mm tolerance range [91]. By implementing Mitchell–Netravali interpolation and RF-based segmentation, our FDM-based approach achieves a mean surface distance of 0.18 mm, effectively meeting these clinical standards at a fraction of the hardware and material cost.

**Figure 10:**
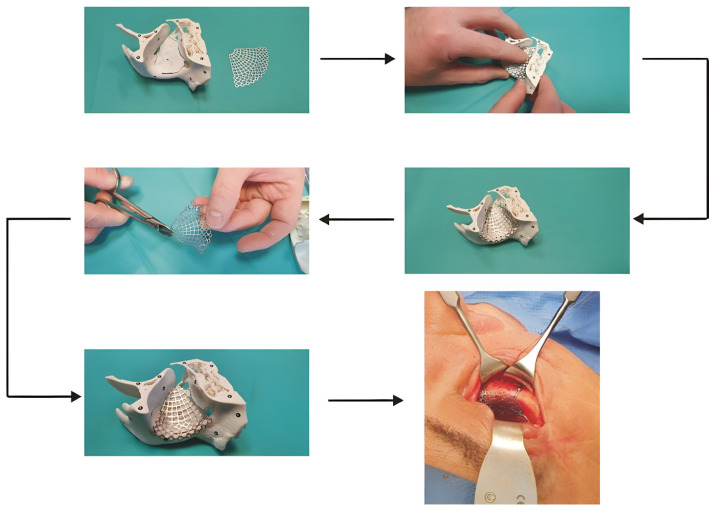
The process of planning and performing a surgical procedure on the developed surgical template for patient no 10.

Models produced using the TARMM procedure were utilised for surgical planning in 21 patients. For instance, Patient No. 10, who suffered a frontal-orbital injury, was treated with a titanium mesh implant. The TARMM-based model was instrumental in delineating the defect area and serving as a template for contouring the titanium mesh (Figure 10).

The surgery was successful, and the patient was discharged on the third postoperative day without complications. Overall analysis revealed that the TARMM procedure significantly reduced the rate of revision surgeries in the orbital region. This improvement is attributed to enhanced surgical precision, as the single-model approach allows surgeons to maintain superior spatial orientation while simultaneously identifying the lesion area and forming the implant. Consequently, the preoperative planning time for models with predefined damage zones was reduced to approximately 30 min.

While these clinical outcomes highlight the efficacy of the proposed method, translating these results into broader surgical practice requires a clear understanding of the workflow’s technical parameters and boundary conditions. Despite the accuracy gains, certain practical limitations of the TARMM workflow must be addressed to guide clinical implementation. First, FDM printing is inherently anisotropic; therefore, the print orientation is critical to ensure that support structures do not compromise the fitting surfaces [92]. We recommend orienting the template so that the anatomical interface is printed with maximum fidelity, ideally away from areas that require dense supports. Second, the thermal stability of standard PLA is a constraint, as its glass transition temperature (Tg = 60 °C) precludes traditional steam autoclaving [93]. For clinical use, low-temperature sterilisation methods, such as hydrogen peroxide plasma, or the transition to high-temperature PLA blends would be necessary [94]. Finally, while TARMM generalises well to high-contrast bony structures such as the mandible or pelvis, its performance in low-contrast environments or extremely small, intricate anatomies (e.g., middle ear ossicles) requires further validation and potentially broader Random Forest training sets to maintain the reported accuracy.

## 4. Conclusions

The study demonstrates that integrating the Template-Assisted Reconstruction of Midface Morphology (TARMM) procedure with Variable Layer Height (VLH) MEX 3D printing significantly improves the quality and efficiency of patient-specific surgical template production. The implementation of the TARMM procedure ensured high geometric fidelity; for the optimised VLH model, the mean dimensional deviation was 0.038 mm (SD ± 0.235 mm), with critical orbital floor areas showing deviations within the range of ±0.1 mm, which is significantly more precise than the nominal CT voxel resolution (0.4 mm × 0.4 mm × 0.7 mm). The results further showed that by using the VLH method (0.3/0.07/0.3 mm), it is possible to reduce manufacturing time by approximately 55%, from 16:32 ± 4:20 [h: mm] for the constant 0.07 mm layer to 7:27 ± 2:26 [h: mm], while simultaneously decreasing material costs from 1.89 to 0.85 per model. Analysis of surface morphology confirmed a very strong linear correlation (r = 0.99, *p* < 0.0001) between layer height and surface roughness parameters, with the VLH strategy achieving an average Sa of 13.06 ± 0.65 μm in critical zones (0.07 mm layer), thereby providing surface quality equivalent to that of high-resolution constant-layer printing. Finally, the proposed workflow was successfully validated in 21 clinical cases, reducing surgeons’ preoperative preparation time to approximately 30 min. This approach provides a cost-effective, precise, and time-efficient solution for patient-specific orbital floor reconstruction, meeting the stringent requirements of modern maxillofacial surgery.

## 5. Patents

The methodology described in this study is based on a patented procedure (Pat. 247185).

## Figures and Tables

**Table 1:** Applied manufactured parameters.

Name of Surgical Guides	3D Printed Area		Variable Parameters (Layer Thickness)
Model_0.3	Without visualising the size of the damage to the orbital area		0.3 mm
Model_0.2			0.2 mm
Model_0.07			0.07 mm
Model_0.3/0.07/0.3			0.3 mm/0.07 mm/0.3 mm
Model_0.3/0.07/0.3_(cut)	With visualizing the size of the damage to the orbital area		0.3 mm/0.07 mm/0.3 mm
**Constant Parameters**			
Ambient temperature: approximately 23 °C (room temperature)		Nozzle temperature: 215 °C for the first layer (to increase fluidity and improve bed adhesion)	
Cooling: constant cooling with 100% fan speed		Nozzle temperature: 210 °C for subsequent layers	
Material: PLA (ROSA3D, Hipolitów, Poland)		Support removal: Mechanical	
Platform temperature: 60 °C		Postprocessing: None	
Filling/type: 80%/grid		Number of contours: 2	
Nozzle diameter: 0.4 mm		A number of dense layers: top—4; bottom—4	
Position in the 3D printer: Central		Movements speed: Contours—70 [mm/s]; filling—200 [mm/s]; bridge—25 [mm/s]	

**Table 2:** Average time and material costs with standard deviation.

	Time [h: min]		Cost [$]	
Model_0.3	4:08 ± 1:04	9:13 ± 2:33	15.62 ± 5.30	34.95 ± 11.85
Model_0.3_(damaged)	5:05 ± 1:29		19.33 ± 6.55	
Model_0.2	5:56 ± 1:43		16.15 ± 4.15	
Model_0.07	16:32 ± 4:20		62.12 ± 15.96	
Model_0.3/0.07/0.3	7:27 ± 2:26		21.58 ± 5.54	
Model_0.3/0.07/0.3_cut	7:26 ± 2:20		20.58 ± 5.20	

**Table 3:** Averaged values of parameters determining the geometrical accuracy of models made using the MEX process.

Whole Model	Number of Points	Maximum Deviation [mm]	Minimum Deviation [mm]	Range [mm]	Mean Deviation [mm]	Standard Deviation [mm]
Model_0.3	307,269	2.852	−1.121	3.973	0.043	0.249
Model_0.2	282,051	2.657	−0.844	3.501	0.040	0.214
Model_0.07	292,617	3.129	−0.744	3.873	0.033	0.231
Model_0.3/0.07/0.3	282,067	2.128	−1.274	3.402	0.011	0.182
Model_0.3/0.07/0.3_(cut)	276,030	3.963	−1.745	5.708	0.093	0.393
**Model fragment**						
Model_0.3	40,147	0.784	−0.472	1.256	0.009	0.100
Model_0.2	33,233	0.720	−0.365	1.085	0.005	0.090
Model_0.07	29,311	1.250	−0.362	1.612	0.029	0.127
Model_0.3/0.07/0.3	27,128	0.690	−0.271	0.961	0.016	0.062
Model_0.3/0.07/0.3_(cut)	27,375	0.597	−0.273	0.870	0.014	0.085

**Table 4:** Selected statistics of the determined surface roughness parameters.

Layer Thickness, mm	Sa			Spk + Sk + Svk,			Rsm		
	Mean [μm]	Std Dev [μm]	CV [%]	Mean [μm]	Std Dev [μm]	CV [%]	Mean [μm]	Std Dev [μm]	CV [%]
0.3	49.52	2.47	4.99	251.68	12.66	5.03	544.83	21.17	3.88
0.2	33.84	1.38	4.09	168.63	20.43	12.12	399.16	56.03	14.04
0.07	16.69	0.85	5.10	100.94	8.33	8.25	228.56	28.10	12.29

**Table 5:** Results of the correlation analysis between layer thickness and surface texture parameters.

Parameter	r-Correlation	Lower 95%	Upper 95%	*p*-Value
Sa	0.9928	0.9649	0.9985	<0.0001
Spk + Sk + Sv	0.9704	0.8613	0.9939	<0.0001
Rsm	0.9728	0.8671	0.9942	0.0003

## Data Availability

The original contributions presented in this study are included in the article/Supplementary Materials. Further inquiries can be directed to the corresponding author.

## Supplementary Materials

The following supporting information can be downloaded at: https://www.mdpi.com/article/10.3390/ma19061208/s1, Figure S1: Data visualization for 21 cases of midface reconstruction.
