# Supplementary figures and images for: Advanced Manufacturing of PLA Surgical Templates for Orbital Floor Geometry: Optimizing Fidelity and Surface Morphology via Variable Layer Height MEX 3D Printing
Source: Materials (Basel). 2026 Mar 19;19(6):1208. doi: 10.3390/ma19061208 (PMC13027635; doi:10.3390/ma19061208)

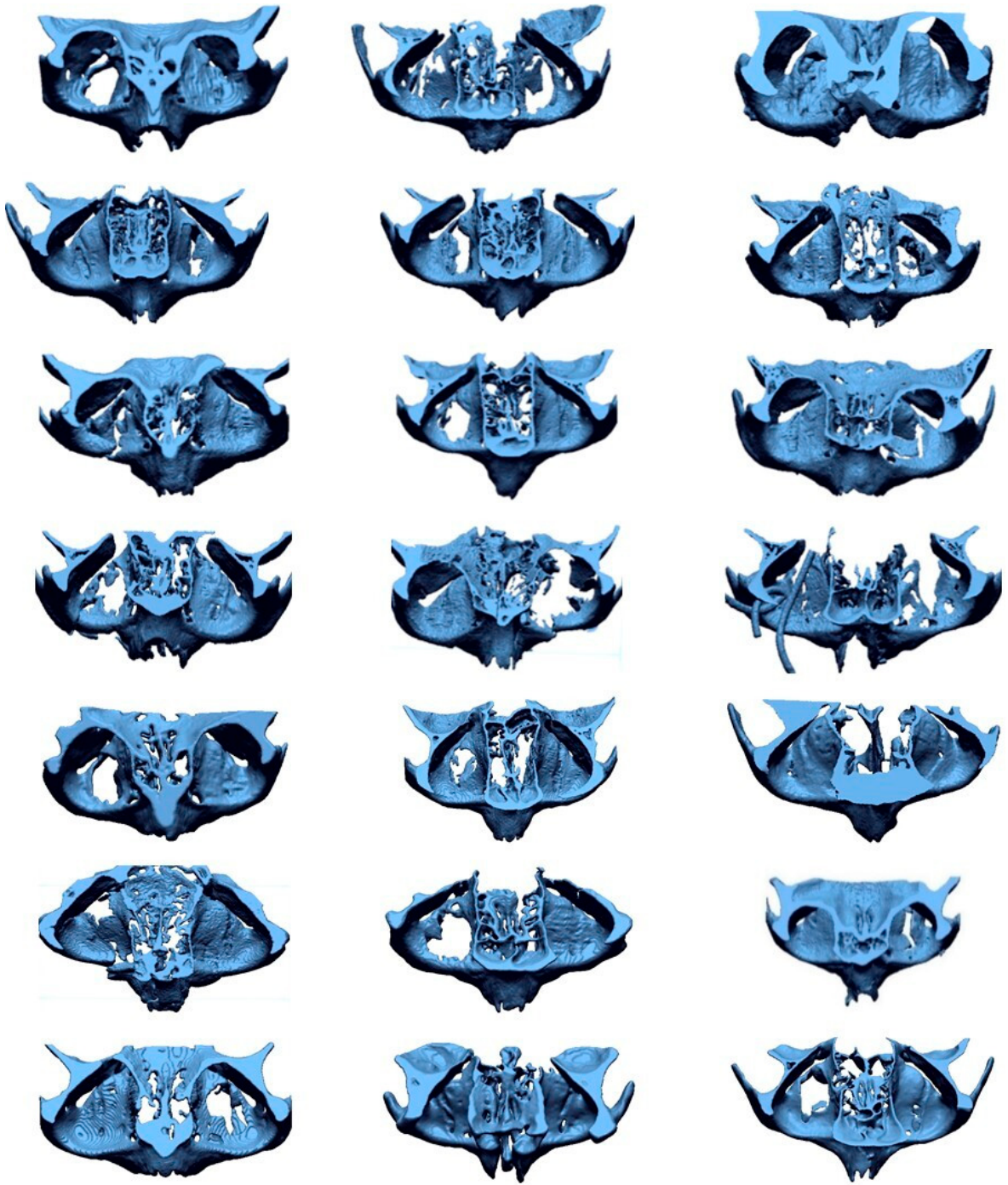

Supplement: Supplementary file 1 [file materials-19-01208-s001.zip › materials-4185282-supplementary.pdf]
